# Supplementary material for: Structure of a heteropolymeric type 4 pilus from a monoderm bacterium
Source: Nat Commun. 2023 Nov 6;14:7143. doi: 10.1038/s41467-023-42872-5 (PMC10628169; doi:10.1038/s41467-023-42872-5)
Supplement: Supplementary file 1 — Supplementary Information [file 41467_2023_42872_MOESM1_ESM.pdf]

Supplementary Information

Structure of a heteropolymeric type 4 pilus from a monoderm bacterium

Robin Anger[1], Laetitia Pieulle[2], Meriam Shahin[3], Odile Valette[2], Hugo Le Guenno[4], Artemis Kosta[4], Vladimir Pelicic[2,3,*], Rémi Fronzes[1,*]

[1]Institut Européen de Chimie et Biologie, Université de Bordeaux-CNRS (UMR 5234), Pessac, France

[2]Laboratoire de Chimie Bactérienne, Institut de Microbiologie de la Méditerranée, Aix-Marseille Université-CNRS (UMR 7283), Marseille, France

[3]MRC Centre for Molecular Bacteriology and Infection, Imperial College London, London, United Kingdom

[4]Plateforme de Microscopie, Institut de Microbiologie de la Méditerranée, Aix-Marseille Université-CNRS, Marseille, France

*Corresponding authors

email: vladimir.pelicic@inserm.fr; remi.fronzes@u-bordeaux.fr

Table S1. Cryo-EM data collection and refinement statistics.

	PDB 8PFB<br>EMDB EMD-17645
**Data collection**	
Microscope	Talos Arctica (ThermoFischer)
Voltage (kV)	200
Camera	K2 Summit (Gatan)
Magnification	45,000 X
Electron dose per frame (e^-/Å^2)	1.34
Electron dose total (e^-/Å^2)	50.74
Pixel size (Å)	0.93
Defocus range (μm)	-0.4 to -1.4
Processing	Single particle analysis
Symmetry imposed	None
Micrographs (no.)	15,025
Initial particle images (no.)	3,022,864
Final particles images (no.)	203,392
Map resolution (Å)	3.67
FSC threshold model	0.143
**Refinement and validation**	
Sharpening *B* factor (Å^2)	104
**Model composition**	
No. of chains	9
Atoms (no.)	17,137
Residues (no.)	1,136
Ligand (no.)	0
Bond lengths (Å)	0.004
Bond angles (°)	0.899
Ramachandran favored (%)	98.45
Ramachandran allowed (%)	1.55
Ramachandran outliers (%)	0
Rotamers outliers (%)	0.32
MolProbity score	1.17
Clashscore	3.85
CC (mask)	0.72
CC (box)	0.26
CC (peaks)	0.25
CC (volume)	0.72

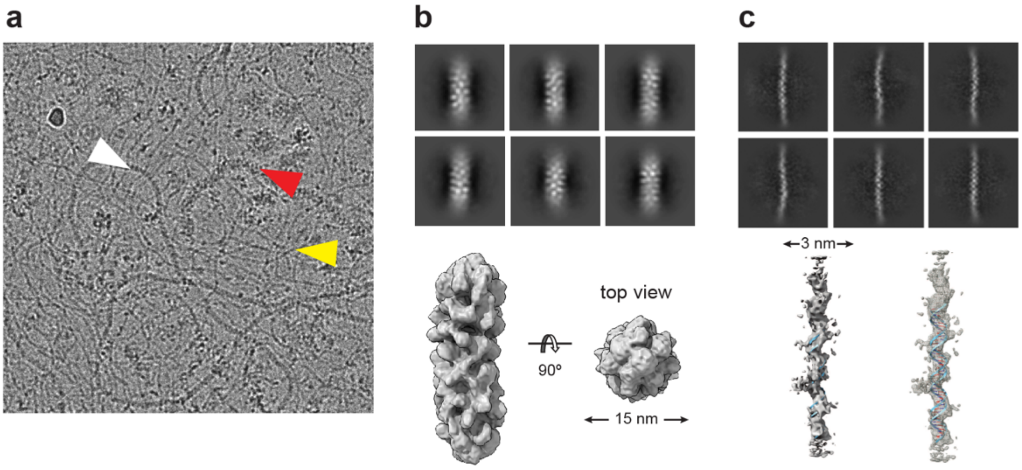

Fig. S1. Filaments in the purified pilus preparations from *S. sanguinis* and their cryo-EM analysis. **a**) Representative cryo-EM micrograph of a purified pilus preparation from *S. sanguinis*. The three types of filaments are indicated by arrowheads: white arrowhead (7 nm-wide filaments corresponding to native T4P), red arrowhead (12 nm-wide "thick" filaments corresponding to T4P), and yellow arrowhead (3 nm-wide "thin" filaments corresponding to DNA). **b**) Analysis of the thick filaments. **Top**, averages after 2D classification. **Bottom**, orthogonal views of the cryo-EM map that was generated. **c**) Analysis of the thin filaments. **Top**, averages after 2D classification. **Bottom**, cryo-EM map in which the B-DNA structure was docked.

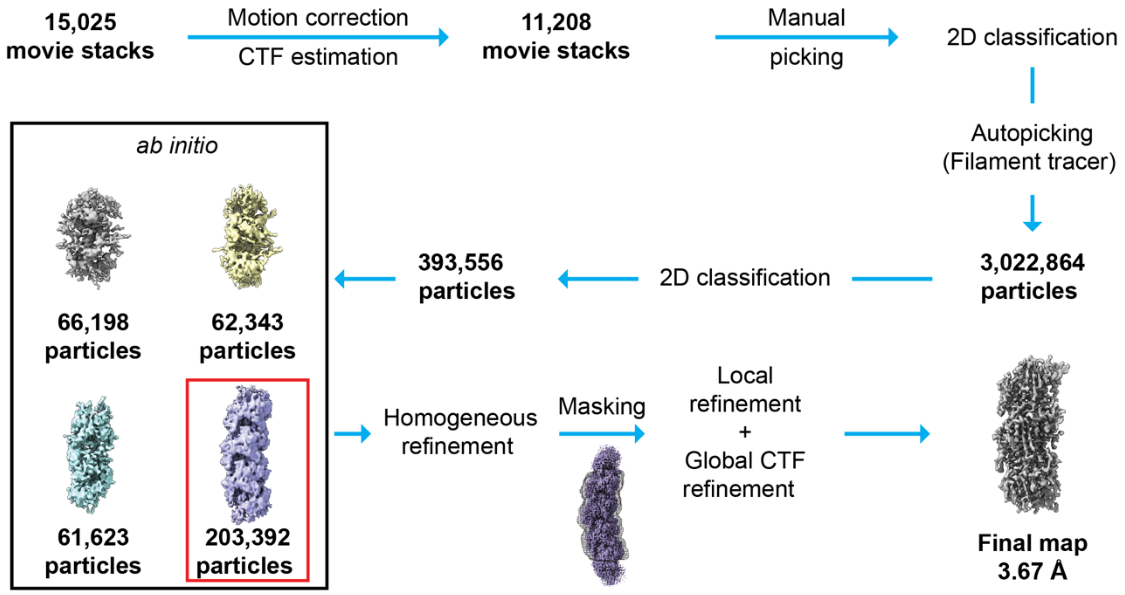

Fig. S2. Cryo-EM data-processing workflow used to generate the final density map obtained in this study. The best *ab initio* model – boxed in red – was used for refinement. See Methods for details.

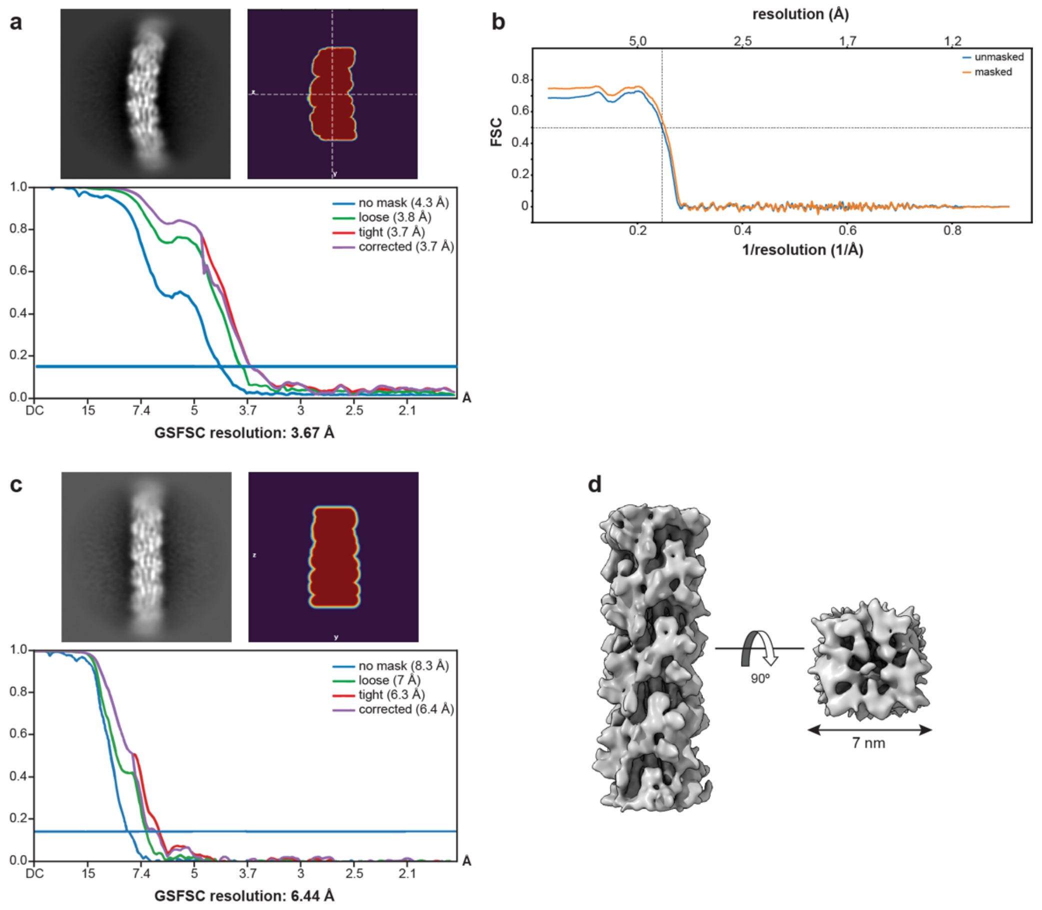

Fig. S3. Cryo-EM maps of *S. sanguinis* T4P obtained with or without imposing a helical symmetry. a) Resolution of the cryo-EM map of *S. sanguinis* T4P obtained without imposing helical symmetry. **Left**, representative average of pilus sections from cryo-EM micrographs after 2D classification. **Right**, slice of the mask used for 3D refinement of the central portion. **Bottom**, estimation of the resolution by gold standard FSC. **b**) Map versus model FSC curves. The map versus model FSC curves were calculated, with and without mask, using Mtriage from Phenix[31]. **c**) Resolution of the cryo-EM map of *S. sanguinis* T4P obtained upon imposition of helical symmetry. **Left**, representative average of pilus sections from cryo-EM micrographs after 2D classification. **Right**, slice of the mask used for 3D refinement of the central portion. **Bottom**, estimation of the resolution by gold standard FSC. **d)** Cryo-EM density map of *S.*

sanguinis T4P with helical symmetry imposition. Side and end views of the filament are presented, with its diameter indicated.

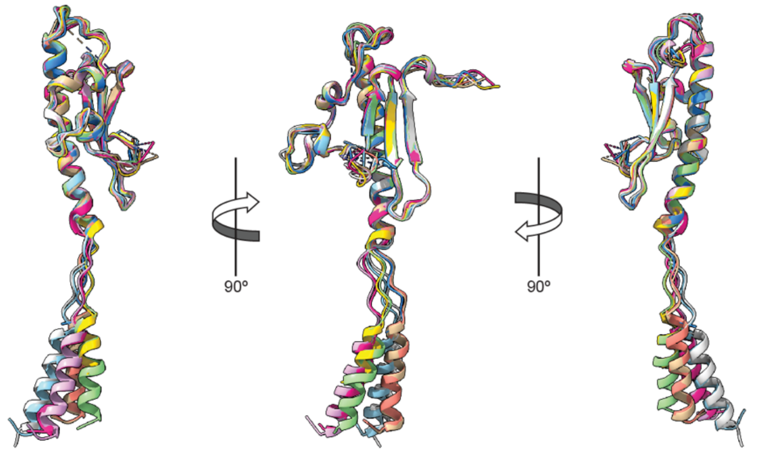

Fig. S4. Flexibility between the different subunits in the *S sanguinis* T4P structure. Superposition of the eight complete pilin subunits, which were resolved in the pilus structure, shows that the pilin globular heads align almost perfectly (1.3 Å RMSD). In contrast, the pilin α1N sticks appear to be intrinsically flexible because they emanate at different angles from the globular heads, thus aligning poorly.

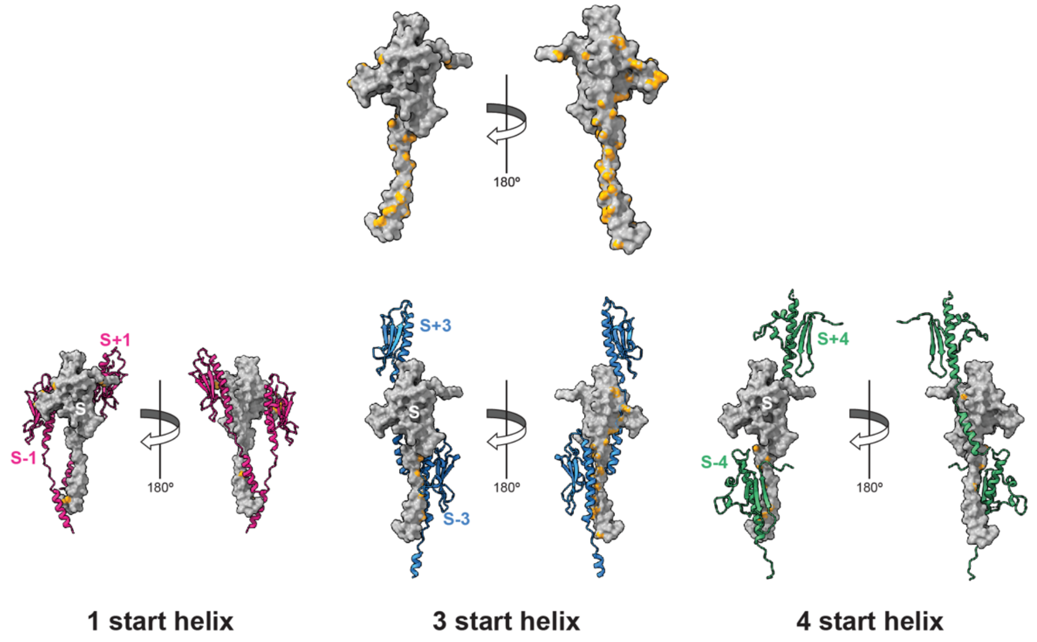

Fig. S5. Areas of interaction between the pilin subunits in the structure of *S. sanguinis* T4P. Top, Surface representation of a pilin subunit located in the central part of the filament shown in two orientations. The interaction areas with the neighbouring subunits are coloured in orange. **Bottom**, interactions between the S subunit and the neighbouring subunits in the 1-start, 3-start and 4-start helices (180º views).
